# Quantifying molecular specificity in excipient-driven antibody solubilization

**DOI:** 10.1080/19420862.2026.2722444

**Published:** 2026-08-29

**Authors:** Zexiang Han, Nadia A. Erkamp, Rob Scrutton, Giuseppe Licari, Olga Predeina, Andreas Evers, Pietro Sormanni, Tuomas P. J. Knowles

**Affiliations:** a Yusuf Hamied Department of Chemistry, University of Cambridge, Cambridge, UK; b Department of Biomedical Engineering, Eindhoven University of Technology, Eindhoven, The Netherlands; c Global Drug Product Development, Biotech Development Center, Merck Serono SA (an Affiliate of Merck KGaA, Darmstadt, Germany), Fenil-sur-Corsier, Switzerland; d Antibody Discovery & Protein Engineering, Merck Healthcare KGaA, Darmstadt, Germany; e Department of Chemical Engineering, Imperial College London, London, UK

**Keywords:** Developability, excipient, formulation, microfluidics, monoclonal antibody, solubility

## Abstract

Excipients are widely used to suppress the self-association of therapeutic proteins, yet their mechanisms of action are not well understood and often assumed to be nonspecific. Here we show that excipient-mediated solubilization of therapeutic antibodies is markedly molecularly specific. Using a high-throughput combinatorial droplet microfluidic platform, we systematically quantify the effects of common pharmaceutical excipients across a diverse panel of monoclonal antibodies (mAbs). Although all studied excipients enhance solubility, their effects can vary significantly between antibodies, spanning dynamic ranges from approximately 7-fold to over 200-fold. Integrating experimental solubilization measurements with sequence- and structure-derived molecular descriptors, we identify interpretable physicochemical determinants underlying excipient responses; for example, the histidine effect is strongly dependent on mAb dipole moment. Our findings reveal trends that highlight the molecular specificity and complexity of antibody–excipient interactions, as well as the limitations of purely generic formulation rules. Overall, this study provides a quantitative framework for analyzing excipient effects across diverse antibodies and supports the development of predictive approaches for rational formulation design. The integration of high-throughput experimentation with molecular feature analysis offers a foundation for improving our understanding and prediction of antibody-specific formulation behavior.

## Introduction

Antibodies have transformed the therapeutic landscape since the turn of the century, representing a potent class of biologic drugs capable of treating or mitigating a range of maladies from infections to cancers. Most antibody drugs approved by regulatory agencies like the US Food and Drug Administration (FDA) are canonical forms of monoclonal antibodies (mAbs), praised for their high specificity, high potency, and favorable safety profiles. Their commercial success is also evident from their substantial and still-growing market size, contributing to a global industry valued at over $200 billion.^1^

Two key and closely related developability traits of antibody-based therapeutics are colloidal stability and solubility.^2,3^ For antibody drugs intended for extensive patient populations or high-dosage regimens, high-yield production and stable formulation of antibodies are critical. Numerous commercial mAbs are formulated at ~50–200 mg mL^−1^ in liquid forms such as in vials or prefilled syringes to be amenable to subcutaneous injection, which enables self-administration and improves patient compliance. Proteins, however, become colloidally unstable at high concentration as short-range attractive interactions begin to dominate, resulting in opalescence and high solution viscosity.^4^ These high-concentration formulations also experience various types of stresses including temperature changes or mechanical agitation, making proteins more susceptible to aggregation which leads to drug inactivation and/or aggregate-mediated immunogenicity.^5^ This constitutes a major challenge in biotechnology and the pharmaceutical industry. One strategy has been to engineer antibody mutational variants to reduce the intrinsic aggregation propensity of drug candidates.^3,6^ Nonetheless, this strategy may impact functionality and can sometimes increase the polyreactivity or the immunogenicity of the drug.^7^

Excipients are inactive substances routinely added to biopharmaceutical formulations to fulfill functionally distinct roles.^8–12^ Essential components include buffers (e.g., histidine, acetate) for pH control and tonicity agents (e.g., NaCl, glycerol, sucrose) for osmolarity adjustment, while others, such as surfactants (e.g., polysorbates) or antimicrobial preservatives (e.g., metacresol), are added depending on the target product profile. Certain excipients, particularly amino acids, salts, and sugars, also function as colloidal stabilizers that improve protein solubility and reduce self-association.^13–17^ Many efforts, both experimental^18,19^ and computational,^20,21^ have been devoted to study protein–excipient interactions in solutions. However, these insights are often derived from simple model proteins like lysozyme or ribonuclease or individual antibodies,^22–24^ limiting their generalizability across different mAb therapeutics.

Therefore, the molecular basis of small‑molecule modulation of the relative solubility of therapeutic mAbs is largely unclarified, and machine learning approaches at the present stage are likely biased due to limited datasets. In particular, knowledge gaps persist regarding the performance of common additives in improving solubility and stability across clinically relevant antibodies. A deeper understanding of antibody formulatability, beyond sequence-level variant optimization, is essential for biologics development.

Herein, we extend a recently developed high-throughput droplet microfluidic platform to investigate whether common pharmaceutical excipients act the same or differentially on the solubility of a panel of therapeutic immunoglobulin G (IgG) antibodies under formulation-relevant conditions. We focus on four widely used additives, histidine, sodium chloride, arginine, and sucrose, and profile their effects across up to 10 clinical-stage mAbs, a panel of notable size and relevance for a mechanistic study. The dense sampling of solubility phase space across excipient concentrations enabled quantitative comparison of excipients in terms of their solubility-enhancing capabilities for a given antibody and across antibodies. Our findings reveal that excipient-mediated solubilization of antibodies is strongly antibody-dependent and can be quantitatively linked to specific molecular features derived from sequence and structure, revealing trends that can support more tailored formulation strategies.

## Results and discussion

### Measuring high-resolution solubility phase diagrams of therapeutic antibodies

The PEG precipitation assay remains a common experimental technique to determine the relative solubility of proteins through molecular crowding and is routinely applied to examine the solution behavior of therapeutic antibodies. Its utility as a predictor of high-concentration solution behavior has been independently demonstrated in multiple studies.^25–27^ Alternative assays, such as affinity-capture self-interaction nanoparticle spectroscopy (AC-SINS), albeit applicable for studying mAb self-association, are less suited for accurately quantifying excipient effects across excipient types.^28^

**Figure 1.**
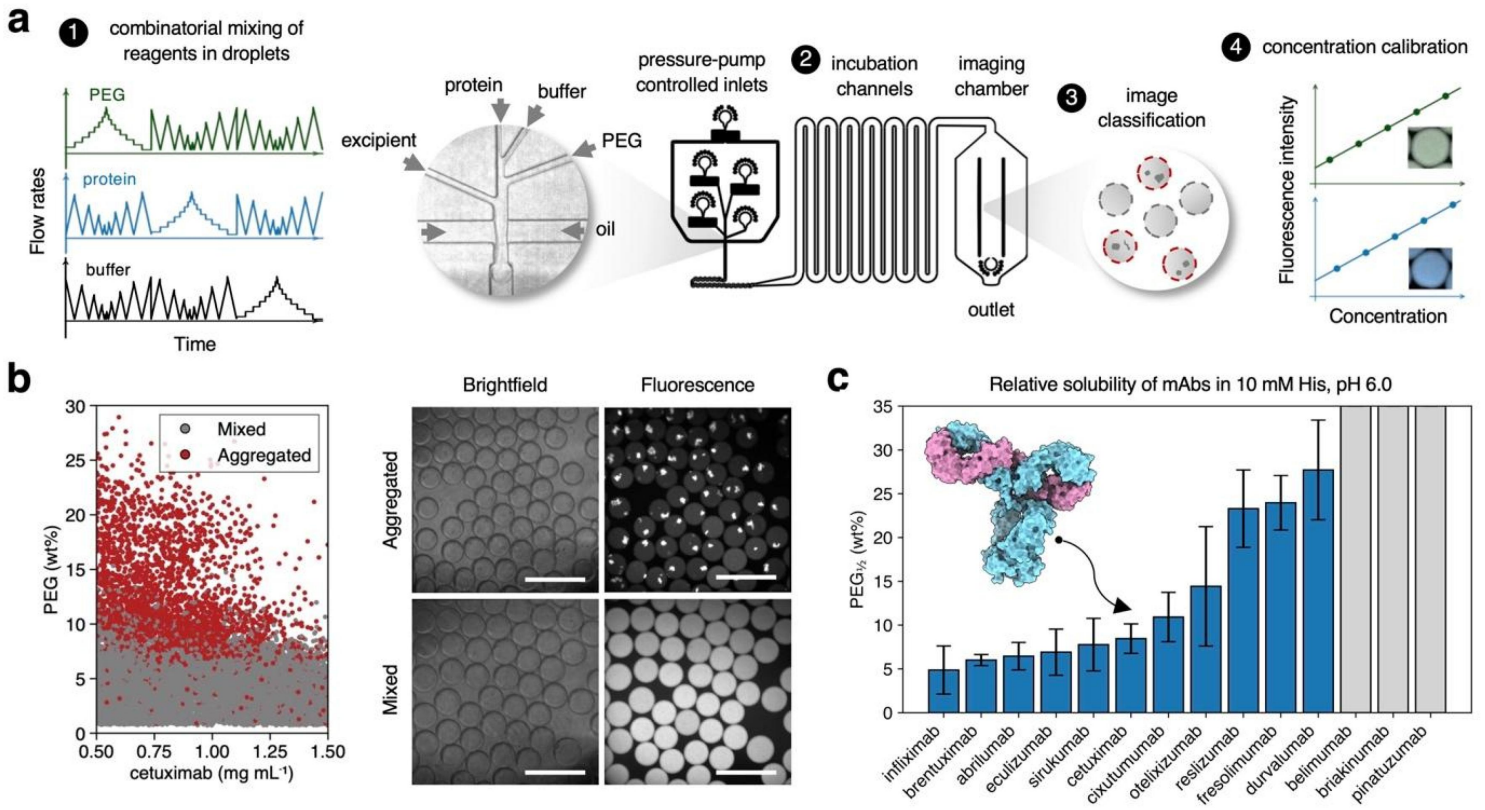
Combinatorial droplet microfluidics for evaluating the relative solubility of clinical antibodies. a, overview of the microfluidic solubility screening assay workflow. (1) combinatorial droplet generation using temporally defined flow profiles. (2) on-chip droplet incubation. (3) droplet imaging and classification. (4) concentration calibration for each ingredient. b, solubility phase diagram of cetuximab around 1 mg mL^−1^ using microfluidic PEG precipitation assay (number of data points *n* = 22,326). Shown on the right are representative images of microdroplets containing proteins (cetuximab) in the precipitated or fully mixed states. “Aggregated” droplets correspond to droplets exhibiting detectable protein-rich aggregates, precipitates, or phase-separated dense structures as identified from fluorescence intensity heterogeneity, whereas “mixed” droplets correspond to visually homogeneous droplets in which no such aggregates were found. c, summary of the relative solubilities of therapeutic antibodies at 1 mg mL^−1^ in standard formulation buffer. The error bars mark the 68% confidence interval as determined from the solubility profiles. Inset shows the full-length cetuximab structural model.

In recent work, we introduced and validated an ultrahigh‑throughput combinatorial droplet microfluidic platform that maps protein solubility and phase behavior across large chemical spaces.^29–31^ The platform is capable of generating thousands of compositionally distinct microdroplets within minutes, each encapsulating different concentrations of protein, PEG, and excipient(s) that serve as a unique chemical microenvironment in which antibody phase behavior can be assessed (Figure 1a).^29,30^ Following on-chip incubation, droplets are imaged by epifluorescence microscopy. Fluorescence intensity measurements are first extracted and used to quantify component concentration on a per-droplet basis. In parallel, droplets are classified in a binary manner based on the presence or absence of optically resolvable phase-separated structures or precipitates in fluorescence images. These two readouts together define the experimental output of the platform. To convert measured fluorescence signals into absolute concentration values, we apply experimentally derived calibration curves that relate fluorescence intensity to protein concentration under assay-matched conditions. This calibration enables accurate reconstruction of the realized droplet compositions and mapping of each droplet onto PEG-protein or PEG-excipient concentration space, from which high-resolution solubility phase diagrams are generated. Given that this approach relies on optical microscopy with a spatial resolution limit of approximately 1 μm, we establish an operational definition of relative solubility for this study: a droplet corresponding to a sample condition is classified as “soluble” if it remains clear of precipitates above this 1 μm threshold. Specifically, relative solubility is defined as the PEG concentration at the precipitation boundary for a fixed mAb concentration of 1 mg mL^−1^, a widely utilized metric to allow direct comparison of solubility across variants and formulation conditions.

Using this microfluidic PEG precipitation assay, we measured the relative solubilities of a panel of 14 IgG1 mAbs (Supplementary File 1), most of which are or are based on marketed and/or clinical-stage drugs, in a standard formulation buffer (10 mM histidine, pH 6.0). Notable features of this technique include an ultralow sample requirement, high-throughput data acquisition, and the ability to directly visualize mAb aggregates as subvisible particles.

The droplet generation process is robust, and each experiment yields a uniform droplet population with a coefficient of variation below 5% (Supplementary Figure S1). Following microdroplet on-chip incubation, solubility classification was performed for the identified droplets. As a representative example, the experiment shown in Figure 1(b) totaled 22,326 data points while using < 50 µg of protein sample. Extrapolating the points of 50% aggregation probability that delineate the phase boundary, we determined the relative solubility of cetuximab at 1 mg mL^−1^ to be PEG_1/2_ = 8.5 ± 1.7 w/w% (Supplementary Figure S2). Owing to efficient mass transport within confined volumes of picoliter droplets, increasing the incubation period beyond 5 min did not lead to significant variations in the measured relative protein solubility, indicating that near-equilibrium conditions were reached within our assay timeframe (Supplementary Figure S3).

Figure 1(c) summarizes the measured relative solubilities of our panel of clinical-stage mAbs, with their solubility profiles shown in Supplementary Figure S4. mAbs with a higher isoelectric point (pI) are generally more electrostatically stabilized in pH 6.0 histidine buffer (Supplementary Figure S5), consistent with previous reports.^32^ Indeed, briakinumab, which because of its high pI has been associated with poor developability metrics such as poly-specificity,^2^ did not precipitate out even for a PEG concentration as high as 30% under the same buffer condition. Given the typical trade-off between antibody binding specificity and self-association,^7^ it could be more desirable to design suitable formulation recipes to prevent antibody aggregation at high concentrations without compromising their intended functional performance (i.e., without increasing the pI too much via mutations). Ten out of the initially considered 14 antibodies with a relative solubility below 25 w/w% PEG were selected for subsequent excipient formulation screening. This threshold was chosen because the microfluidic platform has a practical upper PEG concentration limit of approximately 28.6 w/w% within droplets, which results from the very high viscosity of high-PEG solutions in the microfluidic channels. Antibodies requiring PEG concentrations near this limit to induce precipitation provide insufficient dynamic range to quantify excipient-mediated solubilization effects, thereby hampering comparative analysis across formulations.

### Antibody solubilization by excipients

**Figure 2.**
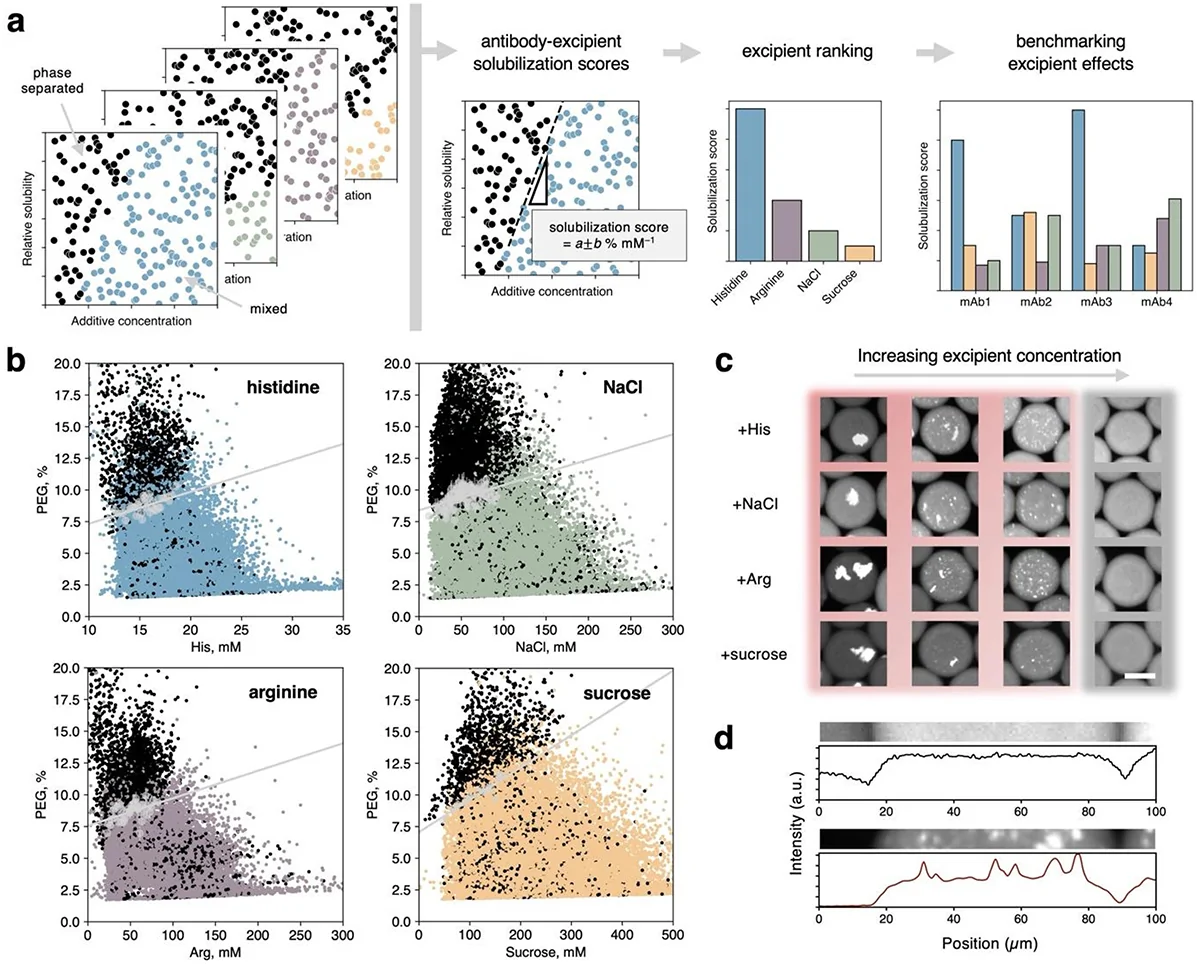
Formulation excipient analysis. a, overview of excipient analysis using high-resolution phase diagrams. b, solubility phase diagram of 1 mg mL^−1^ cetuximab in the presence of common excipients: histidine (*n* = 16,291), sodium chloride (*n* = 21,424), arginine (*n* = 16,491), and sucrose (*n* = 21,702). c, representative images of microdroplets showing antibody solubilization with increasing excipient input. Scale bar = 50 µm. d, fluorescent intensity line profiles for the mixed and phase-separated states.

The selected panel of mAbs, all of which are post‑Phase-I in their development, represents a good set of candidates with confirmed developability for formulation excipient screening. Cetuximab, commercially known as Erbitux, is a marketed drug used to treat colorectal cancer and head and neck cancer.^33^ We selected commonly used formulation additives in approved high-concentration antibody drug formulations, including histidine (His, as a modulator of buffer strength), sodium chloride (NaCl), arginine (Arg), and sucrose to study excipient effects on cetuximab. The concentration range of each excipient was chosen to closely align with those found in approved and marketed products^14^: namely, 10–50 mM His, 50–250 mM NaCl, 50–250 mM Arg, and 100–500 mM sucrose. By examining the phase behavior of fluorescently tagged antibody at a gradient of excipient concentrations, we obtained high-resolution datasets by which excipient performance can be quantitatively compared (Figure 2).

Figure 2(b) illustrates how the relative solubility of 1 mg mL^−1^ cetuximab varies with the addition of four common excipients. The % PEG crowder needed to induce mAb precipitation shifted to higher values with increasing excipient concentration, *i.e.*, excipient addition led to an increase in the relative solubility of the antibody for all four compounds. Our microfluidic screening assay takes advantage of image analysis, allowing for the direct visualization of mAb aggregates, specifically subvisible particles, a feature that is lacking in commonly used bulk measurements. No excipient-specific effects on the mAb aggregate morphology were seen at the micrometer length-scale. Upon PEG precipitation, the antibody exhibited two common precipitated states (Figure 2c). In conditions with no excipients (Figure 1b) or at low concentrations, large, amorphous aggregates were present, featuring irregular and relatively compact structures. In contrast, at higher excipient concentrations, the antibody either formed smaller speckles with diameters of 1–5 µm or remained in a fully mixed state. This range of morphologies reflects the varying phase behavior of the protein across the different conditions surveyed. The precipitated states are protein-rich in nature as they deplete fluorescently labeled mAb molecules from the neighboring environment within microdroplet reaction chambers (i.e., fluorescence is lower outside of the precipitates; Figure 2d).

We applied linear regression analysis to the solubility phase boundary, and the obtained slope was used as a normalized metric for evaluating the excipient’s solubilizing efficiency, generating a *solubilization score* for each antibody–excipient pair (see Methods). A steeper phase boundary slope corresponds to a higher solubilization score, meaning that the same solubility enhancement can be achieved with a smaller increase of molar excipient concentration. To ensure that the solubilization score was not biased by fluctuations in individual fits, we inferred the score using a binning strategy, and slope estimates were obtained across a wide range of minimum-cluster thresholds. The resulting distribution showed a consistent central value, indicating that the solubilization score can be robustly determined (Supplementary Figure S6). For cetuximab, the excipients’ solubilization scores in order of decreasing effect are His (0.266 ± 0.041% mM^−1^), Arg (0.034 ± 0.003% mM^−1^), followed by sucrose (0.024 ± 0.003% mM^−1^) and NaCl (0.023 ± 0.003% mM^−1^) performing similarly.

### Excipient responses can show high variability and molecular specificity

**Figure 3.**
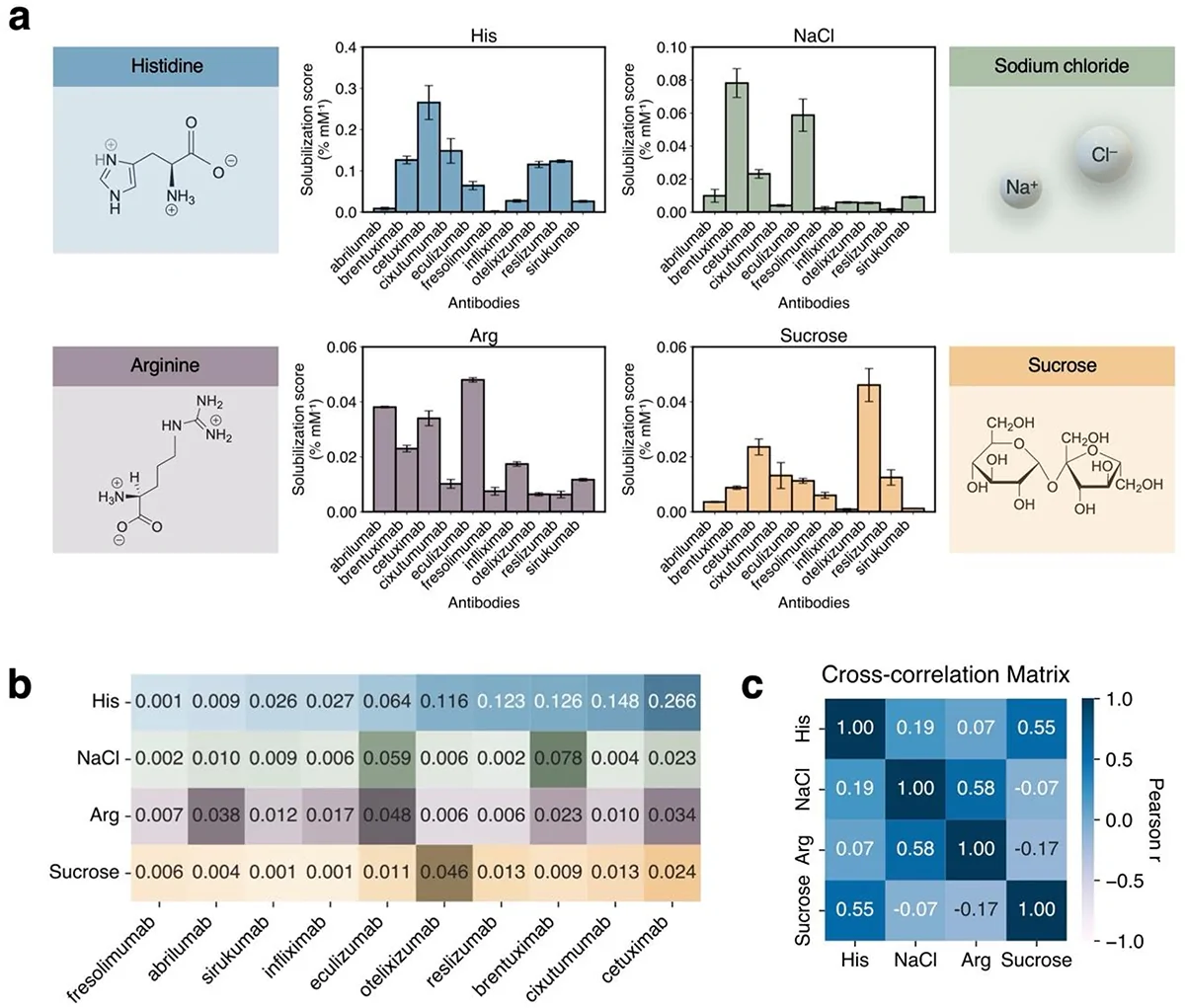
Evaluating solubilization efficiencies of common formulation excipients. a, global comparison of solubilization scores for all protein-excipient pairs characterized. b, heatmap summarizing the data in (a). c, cross-correlation matrix of the excipient solubilization scores based on Pearson correlation coefficient r.

The mechanisms underlying the influence of excipients on protein colloidal stability and solubility remain poorly understood, owing to both the envelope of molecular interactions at play and their dynamic nature. Indeed, biologic formulation design frequently necessitates extensive trial-and-error, or relies heavily on previously approved formulation recipes. To understand excipient-mediated solubilization of clinically relevant antibodies, we established a panel-wide reference by comparing experimentally determined solubilization scores across therapeutic antibodies (Figure 3a).

With a normalized heatmap shown in Figure 3(b), our analysis reveals several key findings regarding the role of excipients on protein relative solubility. Firstly, the solubility-enhancing effects of excipients are often highly antibody-specific. A particularly striking disparity was observed for the histidine buffer: while histidine addition enhanced the relative solubility of cetuximab substantially, the same conferred minimal benefit to fresolimumab, with a greater than 200-fold difference in solubilization scores. This substantial disparity underscores that the effects of small‑molecule additives, including those used as the buffer, are not universally applicable across antibody drugs, even among IgG1 molecules. Secondly, for any given protein, the effects of different excipients can vary substantially, as exemplified by almost all mAbs studied here. Finally, no single excipient universally maximized solubility across the tested antibody panel, indicating that a one-size-fits-all formulation condition for antibody drugs is unlikely. These findings highlight the need for targeted, rational excipient selection to optimize solubility for the therapeutic protein in question. To contextualize these findings, we examined the solubilization efficiency of the excipients on the globular protein bovine serum albumin (BSA), which is smaller and has a simpler surface structure compared to mAbs. We hypothesize that fewer excipient molecules are needed to interact with and stabilize a single BSA molecule compared to an IgG, resulting in higher solubilization scores for BSA. This is indeed observed for all four excipients, albeit to varying extents (Supplementary Figure S7).

Because NaCl, Arg, and sucrose were titrated in a constant 10 mM histidine buffer, we assessed whether excipient responses merely track the histidine baseline across antibodies by examining the cross-correlations of solubilization scores (Figure 3c and Supplementary Figure S8). The scores of NaCl and Arg showed negligible correlation with histidine, indicating that their effects are largely independent of the buffer background and showing mAb specificity. In contrast, sucrose solubilization scores exhibited a strong correlation with histidine across the mAb panel, together with a comparatively constrained dynamic range relative to histidine. These observations suggest that sucrose does not introduce strong antibody-specific modulation under the conditions tested and may instead act through a broadly distributed stabilizing effect that depends on the underlying histidine-driven response landscape. This is consistent with literature descriptions of sucrose as a nonspecific stabilizing excipient, commonly attributed to preferential exclusion and strengthening of the hydrophobic effect.^17,34,35^ Separately, sucrose increased the thermal unfolding transition temperature of cetuximab (Supplementary Figure S9), indicating an overall stabilizing effect on antibody conformational stability.

We additionally note a strong correlation between the solubilization scores of Arg and NaCl (Supplementary Figure S8). However, this co-variation does not imply identical underlying mechanisms, as the two excipients are expected to engage distinct physicochemical interactions (see discussion later).

### Unraveling physicochemical rules dictating excipient responses

Our analysis thus far highlights that excipients do not exhibit a uniform effect on all therapeutic mAbs. We then set out to understand the observed heterogeneity in the solubilization scores between antibody–excipient pairs by examining the physicochemical characteristics of mAbs. To this end, we relate intrinsic antibody physicochemical features to experimentally determined excipient-dependent solubilization responses.

**Figure 4.**
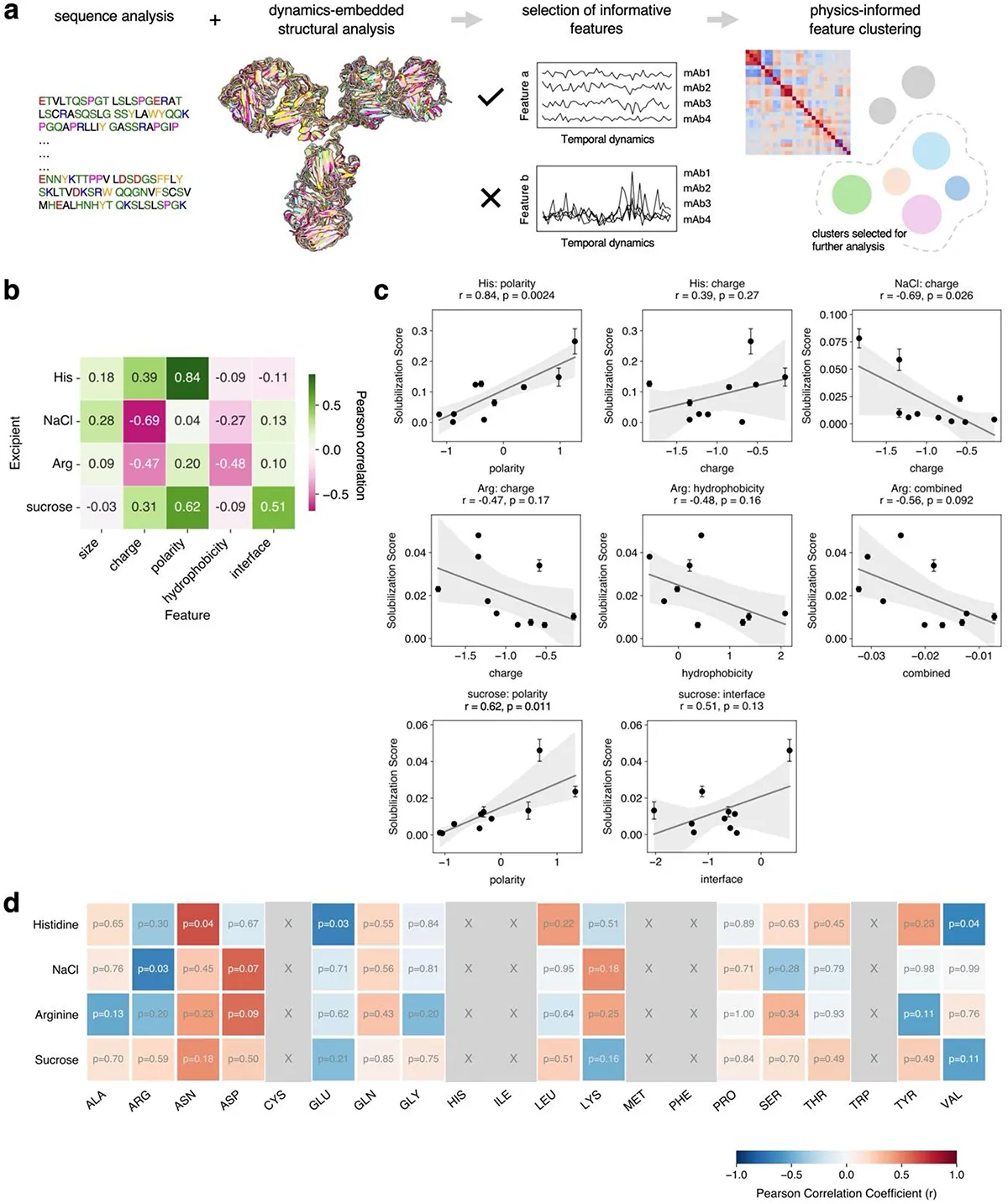
Understanding specific excipient effects on antibody solubility through intrinsic physicochemical profiles. a, physics-based clustering of mAb physicochemical properties using full-length IgG sequence and structural models. Antibody visualization was performed using ChimeraX.^36^ b, heatmap of Pearson correlation coefficients on excipient-specific solubilization scores against feature clusters. c, scatterplots showing the prominent correlation(s) for the four excipients, with linear regression fits and 95% confidence intervals as error bands. Pearson correlation coefficients (r) and associated *p*-values are indicated for each feature. We have also included a scatterplot with Arg solubilization score plotted against a combined score derived from multiple linear regression of both charge (relative weighting = 0.497) and hydrophobicity (relative weighting = 0.503). d, residue-level analysis. Pearson correlation coefficients (r) mapping residue-specific SASAs. Residues with small SASAs and limited cross-panel dynamic ranges were deemed unsuitable for correlation analysis and masked. For the remaining residues, cell color indicates the direction and magnitude of the linear correlation (red, positive; blue, negative), with *p*-values indicated in the corresponding cells. These residue-level correlations are intended to identify potential mechanistic hypotheses.

We first computationally examined a total of 65 clinically relevant mAbs, with substantial overlap with those reported by Jain *et al*.,^2^ including the 14 investigated experimentally in this work. Full-length amino acid sequences were used to compute sequence-level properties. Structural properties were obtained from short atomistic molecular dynamics (MD) simulations employed to relax and improve the quality of structural IgG homology models (see Methods). This procedure generated locally relaxed solution structures that we employed for molecular surface descriptor calculations (Figure 4a), as commonly done in the field.^37–39^ Specifically, some size-related and patchiness metrics were excluded as they were largely invariant across the antibody panel when calculated across the short simulation, reflecting the highly conserved IgG1 architecture and the limited conformational sampling accessible on the simulation timescale employed (Supplementary Figure S10). We retained 19 features for which typical within-antibody fluctuations were small relative to the panel-wide spread, producing a set of sufficiently differentiating features for analysis (Figure 4a). Comparison of key molecular property distributions with those previously reported for marketed and clinical-stage therapeutic antibodies revealed comparable ranges,^12^ placing our dataset within the broader landscape of clinically relevant antibody physicochemical diversity.

The subset studied experimentally largely captures the broader property space of clinically relevant IgG1 antibodies, with the exception of charge-related features (Supplementary Figure S11). Specifically, the selected mAbs were enriched in low-pI antibodies to ensure susceptibility to PEG-induced precipitation under the assay conditions (10 mM histidine, pH 6.0). This constraint was necessary to enable quantitative measurement of excipient effects, as noted previously. Nevertheless, our selected panel spans a broad range of physicochemical properties and provides a representative basis for investigating how molecular attributes influence excipient-mediated solubilization.

The selected features were clustered into five interpretable property categories: size and hydrodynamics, charge, polarity, hydrophobicity, and light-chain/heavy-chain (LC/HC) interfacial properties. Composite z-scores derived from these clusters were subsequently correlated with experimentally measured solubilization scores, revealing molecularly specific stabilization effects of different excipients (Figure 4b).

Histidine is a commonly used amino acid buffer for IgG formulations showing a dual mechanism of action at pH 6, where the zwitterionic His^0^ and protonated His^+^ species co-exist.^40^ This could, in principle, enable the simultaneous contribution of electrostatic screening and imidazole-mediated interactions. While our results were consistent with this interpretation, we found a dominant role of polarity in dictating mAbs’ response to histidine, as measured by dipole moment and polar solvent-accessible surface area (SASA) (Figure 4c and Supplementary Figure S12). Supporting a prevalent role of polarity, residue-level analysis pinpoints tyrosine and asparagine residues of mAbs as potential interaction sites, where mAbs with more exposed polar Asn residues benefit more from the use of histidine for solubilization. The added histidine molecules likely interact via π–π, hydrogen-bonding, and hydrogen–π interactions through the polar imidazole heterocycle to mediate its effects (Figure 4d).^41,42^ Critically, the absence of any significant correlation with hydrophobicity-related metrics suggests that hydrophobic interactions are unlikely to play a dominant role in histidine-mediated stabilization. This indicates that His-mediated stabilization of therapeutic mAbs is fundamentally polar and, to a lesser degree, electrostatic in nature (Supplementary Figure S12), rather than driven by the hydrophobic effect.

Sodium chloride, in stark contrast to the specific interaction of His, operates through nonspecific electrostatic screening. Its ability to solubilize mAbs is uniquely associated with the suppression of electrostatic interactions, as evidenced by strong positive correlations with charge metrics. Intriguingly, at the individual residue level, this screening effect exhibits distinct sensitivities: while the SASA of Asp displays a moderately strong positive correlation with NaCl-mediated solubilization (*r* = 0.59, *p* = 0.075), Glu exhibits a weak, statistically non-significant association (*r* = 0.27, *p* = 0.71). While chemically similar, Glu possesses an additional −CH_2_ group that significantly expands its side-chain conformational space and reduces its local charge density relative to Asp. The higher localized surface charge density of Asp therefore provides a strong electrostatic anchor for interaction with solution ions.^43^ This higher charge density, also observed to drive specific ion interactions in other contexts like biomineralization,^44^ could explain the consistent correlations seen for Asp-NaCl and Asp-Arg compared to Glu. We further note that the investigated antibody panel is biased toward lower-pI species, and the composite charge z-scores are predominantly negative. The observed correlations reflect relative differences in charge within this restricted range rather than the presence of net positively charged antibodies. These findings suggest that NaCl-mediated solubilization is associated with the attenuation of electrostatic interactions that contribute to mAb self-association, with the magnitude of this effect depending on the charge characteristics of individual antibodies.^8,18^

Arginine presents another distinct and specialized mechanism of mAb solubilization. Arg molecules possess a guanidinium group that is positively charged in solution, and in line of this, our analyses identified the nearly equal contribution of electrostatics and protein hydrophobicity in Arg-mediated stabilization (Figure 4c). The guanidinium group of Arg is uniquely suited to form hydrogen bonds with charged regions in antibodies, thereby neutralizing the long-range electrostatic interactions that can drive mAb self-association. On the other hand, the inverse correlation between Arg’s solubilization score and hydrophobic surface area reflects a trade-off in its mechanism. While Arg’s guanidinium group provides potent electrostatic stabilization, its chaotropic character concurrently disrupts the hydrophobic effect and weakens cation–π interactions.^45,46^ In mAbs with larger hydrophobic surfaces, this destabilizing effect offsets a greater portion of the electrostatic benefit, reducing the net solubilization efficiency per Arg added. Specifically, we found Arg effects to depend on hydrophobic SASA, with tyrosine being likely interaction sites (Figure 4d and Supplementary Figure S12). Since the clinical-stage mAbs investigated have good developability properties, their intrinsic stability could buffer the magnitude of this mild destabilization, as separately seen in the reduced thermostability (Supplementary Figure S9).

Finally, the sucrose solubilization score, as measured in histidine buffer, shows a strong correlation with the score for histidine, and their broader correlation profiles share multiple similarities, consistent with its nonspecific stabilization behavior. In fact, the sucrose shows a moderately positive dependency on interfacial stability between the light and heavy chains (Figure 4b and Supplementary Figure S12). This is in agreement with the view that preferential exclusion scales with native state stability, making it easier for sucrose to stabilize mAbs with higher intrinsic interfacial stability.^34^

Overall, we have demonstrated that the effects of common excipients on protein solubility are not promiscuous, even for a system as conserved in molecular attributes as mAbs. Because the excipients are used at much higher concentrations compared to the antibodies, we hypothesize that the excipients form a protein-feature-dependent solvation shell around the proteins, mediating colloidal stabilization.^47^ By combining solubility phase diagrams and in silico physicochemical profiling, we have uncovered physically interpretable correlations between mAb molecular properties and their excipient responses. While some of these relationships are modest in strength, they provide mechanistic insight into the dominant interaction modes engaged by different excipients and can help us understand how competing physicochemical interactions contribute to solubilization.

One limitation of this study is that our analysis relies primarily on linear correlations, which capture only first-order relationships and may not fully reflect nonlinear or higher-order dependencies between molecular features and excipient responses. Additionally, while our mAb panel is notable in its size for excipient analysis, larger, more diverse datasets will be required to fully resolve the molecular determinants of excipient-mediated solubilization and refine predictive modeling. Future investigations should also assess whether the comparative excipient rankings established here translate to other high-concentration, developability-relevant properties, such as viscosity and colloidal interaction parameters like the second virial coefficient. Such complementary measurements will provide deeper insight into the extent to which excipient effects on relative solubility correlate with broader aspects of long-term antibody developability and rational formulation design.

## Conclusions

In this work, we employed a microfluidic PEG precipitation assay for solubility measurements of clinical-stage antibodies and systematic formulation excipient profiling. A key practical advantage of our approach is the collection of high-resolution phase diagrams using minimal amounts of protein, overcoming the material and time constraints of conventional solubility assays. Leveraging the high-resolution datasets, we introduced the solubilization score as a transferable metric for quantifying and systematically comparing excipient performance across proteins. Our data enabled excipient ranking for one IgG and demonstrated that, for a single excipient, its performance is highly variable between IgGs, indicating that formulation additives do not exert universal actions. By integrating phase stability measurements with *in silico* molecular descriptors, we identify the specific physicochemical rules governing excipient responses and map the distinct structural mechanisms underlying these interactions. Rather than exerting uniform effects, we show that excipient performance is highly dependent on antibody-specific features: histidine stabilization is fundamentally polar, correlating with antibody dipole moments; sodium chloride operates via nonspecific electrostatic screening to suppress self-association in low-pI variants; and arginine efficiency reflects a competitive trade-off between electrostatic screening and chaotropic effect. While these relationships vary in strength, they clearly demonstrate that excipient effects are molecularly specific, highlighting the limitations of assuming universal formulation behavior across highly conserved IgG1 frameworks. These results allow us to move beyond a purely empirical paradigm of formulation by providing a mechanistic, physics-based framework that can support the rational design and optimization of antibody formulations. Our work thus complements standard developability studies by shifting the focus from intrinsic aggregation propensities to rational, mechanism-informed formulation design. More broadly, we present a potentially generalizable strategy for quantitatively dissecting small‑molecule-protein interactions, with implications for advancing the physical chemistry of protein and colloidal solutions across biotechnology and medicine.

## Methods

### Materials and antibodies

Polyethylene glycol (PEG, average molecular weight Mw 6000 g mol^−1^; catalog no. 528877), sodium chloride (NaCl), L-histidine, L-arginine hydrochloride, and sucrose were purchased from Sigma‑Aldrich.

IgG1 antibodies were sourced from different pharmaceutical companies or expressed and purified by GenScript. Belimumab, briakinumab, cetuximab, cixutumumab, durvalumab, otelixizumab, and pinatuzumab were provided by the Merck Group. Brentuximab and sirukumab were provided by Novo Nordisk. Infliximab (Catalog no. HY-P9970) was purchased from Cambridge Bioscience Ltd. Abrilumab, eculizumab, fresolimumab, and reslizumab, for which known fragment-variable Fv regions were grafted onto a common IgG1 Fc domain, were acquired from GenScript. Mouse IgG1 (Catalog no. 02-6100) was purchased from Thermo Fisher Scientific. Received antibodies were all purified by affinity chromatography using protein A and were further purified in our lab by size exclusion chromatography (Superdex 200 Increase 10/300 GL column) in 10 mM histidine, pH 6.0, with 150 mM NaCl and buffer-exchanged into 10 mM histidine prior to use. Less than 5 mg of proteins were used in our microfluidic experiments.

### Preparation of microfluidic devices

Polydimethylsiloxane (PDMS)-based microfluidic devices were prepared following the standard soft lithographic protocol. PDMS prepolymer (Sylgard 184 Silicone Elastomer Kit) was mixed with the curing agent in a 10:1 w/w ratio and poured onto the developed silicon master. Degassed PDMS was then cured at 65°C for 2 h. Upon demolding the PDMS slab, inlets and outlets were punched in using a 0.75-mm biopsy punch. Cleaned PDMS was bonded to a glass slide *via* oxygen plasma treatment. The devices were subsequently annealed on a hot plate at 95°C for 5 min, and the microchannel surface was hydrophobically treated with 2% trichloro(1 H,1 H,2 H,2 H-perfluorooctyl)silane (Sigma-Aldrich) in HFE-7500 engineering fluid (Novec^TM^, 3 M). Excess oil was removed with the help of a nitrogen stream before further annealing the device at 95°C for 10 min.

### Combinatorial droplet microfluidics

A custom-built microfluidic platform was used to combinatorially prepare, incubate, and image the microdroplets for solubility screening.^29–31^ The microfluidic device consists of five inlets, a flow-focusing junction, a series of microchannels, and an imaging chamber. Antibodies were fluorescently labeled with Alexa Fluor 488 succinimidyl ester (Thermo Fisher Scientific). PEG solution and excipient solutions were freshly prepared in histidine buffer with appropriate pH adjustment and barcoded with 5 µM Alexa Fluor 647 carboxylic acid and Alexa Fluor 546 carboxylic acid, respectively. Pressure-controlled pumps (OxyGEN, Fluigent) were used to temporally define the flow rates of HFE-7500 oil containing 2% fluorosurfactant (RAN Biotechnologies), antibodies, PEG, buffer, and excipient solutions into the device. Designing suitable flow profiles for each aqueous component enables the combinatorial mixing of PEG, protein, and additives within microdroplets. The oil phase flow rate was kept around 50 μL h^−1^, and the sum of all aqueous flow rates at 105 μL h^−1^. Water-in-oil droplets were generated and confined between two parallel planes, resulting in a discoid shape due to physical compression, with a typical apparent diameter of 80–100 µm. Droplets were incubated for approximately 5 min by flowing through serpentine microchannels to facilitate reagent mixing and continuously imaged using an epifluorescence microscope upon excitations at 488 nm, 561 nm, and 630 nm, respectively.

### Image analysis

Acquired images were analyzed similarly to those previously reported.^29^ Briefly, droplets were identified with a circularity filter and subsequently classified as soluble or aggregate-containing using a pretrained convolutional neural network. Images on phase-separated proteins, including condensates, small speckle assemblies, and large irregular aggregates, were used for data training. For every experiment, a calibration curve of fluorescent intensities *versus* known concentrations for each barcoded component was constructed, and the concentrations of antibodies, PEG, and additives were subsequently determined. This enabled the construction of a solubility phase diagram.

### Calculation of the solubility profile

For each phase diagram, a probability map was generated using a support-vector machine algorithm. The solubility profile for each antibody plots the mAb aggregation probability as a function of PEG concentration at a fixed protein concentration (*i.e.*, 1.0 mg/mL^−1^). To eliminate local stochastic noise, an equal-frequency vertical binning strategy was applied along the PEG axis. Data points were sorted by PEG concentration and binned. For each bin window, the baseline coordinate was defined by the mean PEG concentration, and vertical uncertainty was captured by the standard deviation of the corresponding aggregation probabilities. A sigmoidal function of the form $P\left(x\right)={\left(1+exp\left(-k\left(x-x_{0}\right)\right)\right)}^{-1}$ was fitted using nonlinear least squares regression to the aggregation probability data, where *x* represents the PEG concentration, *x*_*0*_ is the midpoint, and *k* describes the steepness of the curve. Relative solubility was reported as the PEG concentration at 50% aggregation probability, with uncertainty computed as the interval between 16% and 84% probabilities that corresponds to a standard 68% confidence interval centered around the median.

### Calculation of the solubilization score

The solubilization score for each antibody–excipient pair was obtained from high-resolution single-droplet phase diagrams as the slope of the inferred phase boundary using a similar binning-based classification procedure. Adaptive bins were constructed such that each bin contained at least a specified minimum number (minN) of both phase-separated and mixed droplets. This aggregation stabilized the local classification problem by ensuring that each bin contained sufficient information from both classes using balanced accuracy. The PEG value yielding the highest balanced accuracy was assigned as the local phase boundary, and bins with accuracy < 0.75 were excluded. Average phase-boundary slope and corresponding standard deviation were computed over minN = 30–100.

### Thermostability measurements

The thermal stability of antibodies was evaluated via intrinsic fluorescence measurements using the NanoTemper Tycho NT.6 system using an excitation wavelength of 280 nm. Proteins were formulated at 1 mg mL^−1^ in 10 mM His buffer at pH 6.0, and separately, with the addition of excipients. Samples were loaded into Tycho glass capillaries and subjected to a heating protocol from 35°C to 95°C at a rate of 0.5°C s^−1^. The first derivative of the resulting fluorescence ratio curve was calculated. The onset temperature of the thermal denaturation T_onset_ was calculated as the temperature at which the first derivative showed a detectable deviation from the baseline, with the threshold set to 10^−3^. Measurements were performed in duplicates.

### Modeling full-length antibodies

A total of 65 clinically relevant antibodies were considered, including those investigated experimentally in this work. Among them, 43 are approved and marketed products, 8 are in clinical development as of 2026, 11 have been discontinued, and the remaining 3 are associated with antibody-drug conjugates that are either approved or currently undergoing clinical evaluation. The full-length antibody sequences are provided in Supplementary File 1. We constructed full-length antibody homology models using the Antibody Modeler module of Molecular Operating Environment (MOE) 2022.02.^48^ The Amber10:EHT force field was employed in combination with the Generalized Born implicit solvent model,^49^ with internal and external dielectric constants set to 4 and 80, respectively. Nonbonded interactions were smoothly switched off between 10 and 12 Å. All N- and C-termini were retained in their standard, charged forms. Following model generation, structures were prepared in MOE to relieve steric clashes and to assign protonation states at pH 6.0 using the Protonate3D protocol.^50^ Final models were energy minimized until the energy gradient was below 10^−2^ kcal/mol·Å^2^.

### Molecular dynamics simulations

For classical atomistic molecular dynamics (MD) simulations, the homology models were embedded in a rectangular water box with at least 20 Å between any protein atom and the box boundary, and neutralized with NaCl using Visual Molecular Dynamics (VMD).^51^ Equilibration runs were performed with Nanoscale Molecular Dynamics (NAMD) 2.14, whereas production simulations were carried out with NAMD 3.0b6.^52^ The CHARMM36m force field^53^ was used to describe the protein, and TIP3P water^54^ was used for solvation. Nonbonded interactions were computed with a 12 Å cutoff and a switching function starting at 10 Å. Long-range electrostatics were treated using the Particle Mesh Ewald (PME) method,^55^ with a grid spacing of 1 Å^−3^. All bonds involving hydrogen atoms were constrained using the SHAKE algorithm.^56^ Pressure was maintained at 1 atm via a modified Nosé–Hoover–Langevin piston,^57^ and the temperature was controlled at 300 K with a Langevin thermostat. A timestep of 2 fs was used, evaluating Lennard–Jones interactions at every timestep and updating PME forces every second timestep.

Antibody equilibration followed a 3-step protocol: (1) 2000 steps of conjugate-gradient energy minimization, (2) a 200-ps NPT run with a temperature ramp from 0 K to 300 K, and (3) an additional 200‑ps NPT simulation at 300 K. The final snapshot from stage (iii) provided starting coordinates and velocities for production trajectories of 30 ns per antibody. Trajectory analysis was carried out in VMD, extracting 50 frames from the last 24 ns of each simulation after recentering and aligning the protein within the simulation box.

We note that 30 ns simulations are not intended to sample slow, large-amplitude conformational dynamics such as inter-domain rearrangements or hinge flexibility, which occur on much longer timescales. Rather, the simulations serve to provide time-averaged, single-molecule physicochemical descriptors that capture local conformational fluctuations of solvent-exposed residues, which are relevant to excipient interactions and equilibrate well within the simulated timescale.

### In silico physicochemical profiling of antibodies

Physicochemical descriptors were calculated from full-length antibody sequences and homology models. Expasy ProtParam was used to calculate the number of residues, molecular weight, isoelectric point, aliphatic index, and GRAVY (grand average of hydropathy) scores of the antibodies. Solvent-accessible surface areas (SASAs) were computed for categorized residues: hydrophobic (ALA, VAL, LEU, ILE, PRO, PHE, MET, TYR, TRP), aliphatic (ALA, VAL, LEU, ILE), aromatic (PHE, TYR, TRP), polar (SER, THR, ASN, GLN, TYR), positively charged (ARG, HIS, LYS), and negatively charged (ASP, GLU). Histidine protonation at pH 6.0 was modeled with 50% occupancy. Surface patches were identified using DBSCAN clustering of exposed residues (SASA > 0.1 nm^2^), with mean patch areas calculated for each residue category. Additional parameters were directly calculated from the MOE, including net charge, radius of gyration, sedimentation coefficient, dipole moment, affinity between light and heavy chains (affinity HC/LC), and buried surface area between light and heavy chains (BSA HC/LC). All structure-based properties were averaged across 50 frames to account for conformational dynamics of proteins in solution.

Feature selection and clustering. Five feature clusters were considered including size, charge, polarity, hydrophobicity, and interfacial properties. Properties within the size and hydrodynamic cluster include number of amino acid residues, molecular weight, and protein volume. Charge concerns isoelectric point (pI), net charge at pH 6, variable-domain Fv net charge, fraction of positively/negatively charged residues, and positively/negatively charged SASAs. Polarity concerns dipole moment and polar SASA. Hydrophobicity concerns aliphatic index, GRAVY score, and hydrophobic/aromatic/aliphatic SASAs. Interfacial properties concern affinity between HC/LC and buried surface area between HC/LC.

Features within each cluster were z-score normalized and physically aligned such that higher values uniformly represent increased property magnitude (e.g., a higher Charge composite score indicates a more positive character). Specifically, composite scores were calculated as equal-weighted averages of aligned z-scores, i.e., $C_{k}=\frac{1}{n}\overset{\;}{\sum }a_{j}z_{j}$, where $z_{j}$ are z-scored feature *j* and $a_{j}=\pm 1$ align features to uniform directions. This yielded five interpretable metrics for each antibody.

## Supplementary Material

Supplemental Material

Supplementary File 1 antibody seqs.txt

## Data Availability

The authors confirm that the data supporting the findings of this study are available within the article and its supplementary materials. Additional datasets generated during this study, including in silico homology structural models, their molecular dynamics snapshots, and associated analysis files, are available from Zenodo at https://doi.org/10.5281/zenodo.21836254.
