# Supplementary material for: Quantifying molecular specificity in excipient-driven antibody solubilization
Source: MAbs. 2026 Aug 29;18(1):2722444. doi: 10.1080/19420862.2026.2722444 (PMC13532009; doi:10.1080/19420862.2026.2722444)
Supplement: Supplementary file 1 [file KMAB_A_2722444_SM1083.docx]

**Supplementary Information**

**Quantifying molecular specificity in excipient-driven antibody solubilization**

Zexiang Han *et al.*

**Supplementary Figure S1. Size distribution of water-in-oil microdroplets.** **a**, Apparent microdroplet size distribution histograms sampled from 23 representative microfluidic experiments. Note that while the mean droplet size can vary slightly between experiments, the measured phase boundary has been shown to be droplet volume-insensitive.^29^ **b**, Table summarizing the number of droplets analyzed, apparent mean microdroplet radius in µm, standard deviation, coefficient of variation (CV) and polydispersity index (PDI).

**Supplementary Figure S2. Determination of the solubility profile of an antibody.** **a**, Relative solubility phase diagram of cetuximab with binary classification of data points, as shown in Figure 1b. **b**, Averaged solubility phase diagram, where local aggregation probability was computed by spatial averaging of neighboring measurements within the composition space. The averaged representation highlights the underlying phase boundary while preserving the experimentally sampled composition range. wt% means w/w%. **c**, Aggregation probabilities at 1 mg mL^−1^ for various PEG concentrations were extrapolated from the phase diagram, and a sigmoidal fit was used to calculate antibody relative solubility as the PEG concentration for which the aggregation probability is 50%. Here, the relative solubility of cetuximab was determined to be 8.5±1.7 w/w%.

**Supplementary Figure S3. Effect of microdroplet incubation time on the measured antibody solubility. a,** Measured solubility phase diagrams of a model mouse IgG1 antibody following increasing durations of on-chip or on-line incubation. **b**, Corresponding sigmoidal fits and the determined relative solubilities at 1 mg mL^−1^. The phase boundaries show insignificant variations with time. These results illustrate the robustness of the screening method, where changes in incubation time beyond 5 min minimally affect the solubility profile. This is likely generalizable to the IgG antibodies studied in this work.

**Supplementary Figure S4. Measured solubility profiles of a panel of therapeutic antibodies.** **a**, Solubility profiles of 11 antibodies with sigmoidal fits. **b**, Phase diagrams of three mAbs (belimumab, briakinumab, pinatuzumab) that did not precipitate out under the assay conditions, showing only mixed phase.

**Supplementary Figure S5. Correlation between mAb relative solubility and its charge.** **a,** Calculated full-length isoelectric point. **b,** Calculated charge at pH 6.0.

**Supplementary Figure S6. Calculation of solubilization scores. a,** Cetuximab-NaCl phase diagrams showing the extrapolation of phase boundary slope using different binning size minN = 50, 100, and 150. **b-e,** Fitted slope as a function of the minimum cluster size applied to (b) His, (c) NaCl, (d) Arg, and (e) sucrose.

**Supplementary Figure S7. Excipient effects on bovine serum albumin (BSA) in standard formulation buffer. a,** Protein structure of BSA (PDB 3V03) compared to an IgG protein cetuximab. **b,** Solubility phase diagrams of BSA in the presence of histidine (*n =* 36,163), sodium chloride (*n =* 27,892), arginine (*n =* 30,495), and sucrose (*n =* 41,080) and determined solubilization scores.

**Supplementary Figure S8. Scatterplots showing the cross-correlations of solubilization scores.** Units in % mM^−1^. Inset shows the cross-correlation matrix of the excipient solubilization scores based on Spearman’s rank correlation coefficient.

**Supplementary Figure S9. Thermostability analysis. a,** Thermal unfolding curves of cetuximab in the absence vs. presence of formulation excipients at different concentrations. Measurements were taken in duplicates for each condition, shown in solid and dashed lines, respectively. **b,** Effect of excipient addition on the onset temperature T_onset_ of mAb. The dashed line indicates the thermal unfolding transition temperature measured in the absence of excipient and is shown to facilitate comparison with excipient-containing samples.

**Supplementary Figure S10**. **Identification of differentiating physicochemical features.** Features that are sufficiently differentiating across the antibody panel were retained for subsequent analysis. The remaining ones, notably patchiness-related metrics, were excluded. Please see Methods for feature descriptions/definitions.

**Supplementary Figure S11. mAb diversity and representativeness.** (a) Violin plots showing the distribution of composite z-scores for each feature cluster across 65 clinically relevant mAbs. Dashed and solid lines mark the 75^th^, 50^th^ and 25^th^ percentiles, respectively. The 14 experimentally investigated mAbs are highlighted: 10 mAbs that were precipitable and used for excipient analysis are shown in circles, with the remaining 4 mAbs shown in triangles. (b) Violin plots showing the distributions of selected, individual molecular features computed for the same set of mAbs. The selected subset mostly captures the broader property space of clinically-relevant IgG1 antibodies, except for charge-related features, supporting feature diversity and the representativeness of the selected mAb panel. Notably, mAbs with relatively low isoelectric points (pI) were used for experimental studies, as reduced electrostatic stabilization facilitates PEG-induced precipitation thus enabling the experimental evaluation of excipient effects.

**Supplementary Figure S12. Correlation analysis against specific mAb molecular features.** Please see Methods for feature descriptions/definitions.
